# IgG from Adult Atopic Dermatitis (AD) Patients Induces Nonatopic Neonatal Thymic Gamma–Delta T Cells (γδT) to Acquire IL-22/IL-17 Secretion Profile with Skin-Homing Properties and Epigenetic Implications Mediated by miRNA

**DOI:** 10.3390/ijms23126872

**Published:** 2022-06-20

**Authors:** Beatriz Oliveira Fagundes, Thamires Rodrigues de Sousa, Andrezza Nascimento, Lorena Abreu Fernandes, Fábio da Ressureição Sgnotto, Raquel Leão Orfali, Valéria Aoki, Alberto José da Silva Duarte, Sabri Saeed Sanabani, Jefferson Russo Victor

**Affiliations:** 1 Laboratory of Medical Investigation LIM-56, Division of Dermatology, Medical School, University of Sao Paulo, Sao Paulo 05403-000, Brazil; bibifags73@gmail.com (B.O.F.); sousarthamires@gmail.com (T.R.d.S.); raquelleao@hotmail.com (R.L.O.); valeria.aoki@gmail.com (V.A.); adjsduar@usp.br (A.J.d.S.D.); 2 Post-Graduation Program in Translational Medicine, Federal University of Sao Paulo, Sao Paulo 04039-002, Brazil; andrezza.ns@gmail.com (A.N.); lorena.abreu.fernandes@gmail.com (L.A.F.); 3 Division of Hematology, Medical School, University of Sao Paulo, Sao Paulo 05403-000, Brazil; fabio.house@hotmail.com; 4 Division of Pathology, Medical School, University of Sao Paulo, Sao Paulo 05403-000, Brazil; 5 Laboratory of Medical Investigation LIM-03, Division of Pathology, Medical School, University of Sao Paulo, Sao Paulo 05403-000, Brazil; 6 Faculdades Metropolitanas Unidas (FMU), Health Sciences School, Sao Paulo 04505-002, Brazil; 7 Medical School, Universidade Santo Amaro (UNISA), Sao Paulo 04829-300, Brazil

**Keywords:** atopic dermatitis, IgG, IL-22, p-bodies, γδT cells, thymus, human, IL-17, miRNA

## Abstract

γδT cells mature in the human thymus, and mainly produce IL-17A or IFN-γ, but can also produce IL-22 and modulate a variety of immune responses. Here, we aimed to evaluate whether IgG from AD patients (AD IgG) can functionally modulate thymic nonatopic γδT cells. Thymic tissues were obtained from 12 infants who had not had an atopic history. Thymocytes were cultured in mock condition, or in the presence of either AD IgG or therapeutic intravenous IgG (IVIg). Following these treatments, intracellular cytokine production, phenotype, and microRNA expression profiles were investigated. AD IgG could downregulate α4β7, upregulate CLA, and induce the production of IFN-γ, IL-17, and IL-22 in γδT cells. Although both AD IgG and IVIg could directly interact with γδT cell membranes, AD IgG could reduce γδT cell apoptosis. AD IgG could upregulate nine miRNAs compared to IVIg, and six when compared to the mock condition. In parallel, some miRNAs were downregulated. Target gene prediction and functional analysis indicated that some target genes were enriched in the negative regulation of cellular transcription. This study shows that AD IgG influences the production of IL-17 and IL-22 by intrathymic nonatopic γδT cells, and demonstrates epigenetic implications mediated by miRNAs.

## 1. Introduction

γδT cells are a minority subset of T cells found in the peripheral organs and blood [1]. These cells express T-cell receptors composed of γ and δ chains (γδTCRs), and their ligands are still unknown, as recently discussed [2,3,4,5]. γδT cells can mediate the human atopic state [6,7,8], and some researchers suggest that they are involved in the development of atopic dermatitis (AD) [9,10,11,12]. Because of the functional plasticity of γδT cells, which are typically divided into IL-17- and IFN-γ-producing γδT cells, γδT cells can also produce other cytokines with modulatory effects, such as IL-22 [13].

Human peripheral γδT cells coproduce IL-17 and IL-22 at a low frequency [14]. The same study revealed that the conditions that favored the production of IL-17A by γδT cells also favored the production of IL-22, a feature that remains unknown, especially in the context of skin diseases, since IL-22 production is instrumental in the maintenance of skin homeostasis [15], as evidenced in AD development [16,17,18] and treatment [19].

In recent years, there has been debate about IgG molecules acting as natural ligands of lymphocytes and modulating their functional properties on the basis of the immunological status of IgG donors [20,21,22]. On the basis of murine observations that maternal IgG inhibited offspring allergy [23,24], some translational approaches have revealed that peripheral B cells can be modulated to acquire regulatory functions by secreting IL-10 in response to IgG from nonatopic donors [25,26]. Thymic and peripheral αβT cells’ cytokine production may also be modulated by the donors’ atopic state. In these studies, IgG from atopic individuals could modulate the production of IFN-γ by TCD4 and TCD8 cells [27], IgG from AD patients modulated the production of IL-17 and IL-10 by TCD4 and TCD8 cells [28], and IgG from HIV-1-exposed noninfected individuals modulated the production of IFN-γ by thymic TCD4 and TCD8 cells [29].

Other studies have shown that IgG from atopic or AD patients modulates thymic innate lymphoid cell subsets (ILC1, ILC2, and ILC3) [30] and thymic iNKT cells [31]. The effect was demonstrated in both studies by modulating cytokine production.

In terms of γδT cells, purified IgG could also modulate γδT cells. This effect was demonstrated in a translational study that illustrates the IgG-mediated regulation of thymic γδT cells IL-17 production, with some possible implications for allergy development [32], and also in a human approach where IgG from nonatopic individuals can modulate the production of IFN-γ and IL-10 on thymic and peripheral γδT cells [33].

Together, all of the aforementioned experimental approaches failed to elucidate the mechanism by which IgG interacts with and precisely modulates T- and B-cell functions. However, they all suggest that this effect may be more prominent on neonatal thymic cells and that IgG may directly interact with γδT cells to mediate the modulatory effect.

γδTCR signaling is described as critical in determining the functional activity of γδT cells, mainly determining the thymic development of two central γδT cell subsets characterized by the production of IL-17A or IFN-γ [34]. The natural recognition of γδTCR or other membrane receptors by IgG is not described in the literature, but given the evidence that IgG can directly modulate thymic T cells, including γδT cells, these interactions may occur and mediate some functional modulation.

Furthermore, it became an essential point of investigation whether the effect of IgG-interaction with γδT cells can induce molecular alterations that result in functional modulation. This approach can be addressed by evaluating miRNA expression in these cells. MiRNAs are short noncoding RNAs of approximately 22 nucleotides in length that can modulate the expression of target genes through binding to their messenger RNAs (mRNAs) and triggering mRNA degradation, thereby inhibiting the molecular expression of the targeted gene [35,36].

This epigenetic mechanism was demonstrated to regulate T and B cells’ functions, including the maturation and function of lymphocytes [37,38]. Several studies have found that miRNA expression is significantly dysregulated in inflammatory disorders, such as AD [39]. Furthermore, in a murine model of atopic disease, a recent study from our group revealed that the thymic functional modulation of IL-17-producing γδT cells had epigenetic implications, as evidenced by the modulation of miRNAs expression on neonatal thymus [40].

On the basis of the information presented above, we first sought to determine whether IgG molecules purified from AD patients could regulate the functional properties of γδT cells, whether IgG molecules could directly interact with γδT cell membranes, and whether this interaction could exert functional modulation with some epigenetic implication by controlling the expression of miRNAs.

## 2. Methods

### 2.1. Patient Samples

Thymic tissues were obtained from 12 neonate patients aged less than 7 days (3.4 ± 0.54 days), and six infant patients aged 5 to 6 months (5.4 ± 0.23 months) who had undergone corrective cardiac surgery at the Hospital do Coração (HCor) Sao Paulo, Sao Paulo, Brazil. Patients were required to meet the following criteria: no immunodeficiency, no genetic syndromes or allergic reactions, and no immunosuppressants. The parental history of allergic disease was reviewed, and only children of nonatopic parents were included in this study.

Similar to previous studies performed by our group [28,31], we recruited 14 adult patients diagnosed with AD according to Hanifin and Rajka’s criteria, and clinically categorized them as moderate or severe using the eczema area and severity index (EASI) [41]. There were eight male patients and six female patients. The patients’ ages ranged from 24 to 35 years. The average disease duration was 26 years. The selected patients were admitted to the study during a scheduled visit to the Dermatology Outpatient Clinic service at the University of Sao Paulo, Sao Paulo, Brazil. None of the patients was given systemic corticosteroids (intravenous, oral, or potent topical) or immunosuppressants for at least four weeks. The health controls (HC) group included 23 clinically diagnosed non-AD volunteers aged 20 to 40 years.

Each thymus was obtained from a different donor, and the results were obtained through six independent experiments. This study was approved by the HCor and the School of Medicine ethics committees at the University of Sao Paulo (CAAE: 15507613.4.0000.0060).

### 2.2. Thymic Tissue Dissociation, Cell Isolation, and Storage

Thymocytes were released from the tissue samples using enzymatic dissociation as previously described [42]. The thymus was divided into small fragments and transferred to conical centrifuge tubes containing RPMI medium, 0.5 mg/mL collagenase A and 0.02 mg/mL DNase I (Roche Diagnostics, Mannheim, Germany), as previously standardized by our group [28]. The digested fragments were homogenized, filtered through a plastic sieve to remove aggregates, and washed with the resulting cell suspensions. The cells were then resuspended, and the low-density fraction was collected via Ficoll gradient centrifugation (GE Healthcare Bio-Science, Uppsala, Sweden). The thymic cells were snap-frozen and kept in liquid nitrogen until needed.

### 2.3. IgG Purification, Isotypes Evaluation, and Labeling

As previously described, IgG purification from pooled sera from HC and AD was performed using the Melon Gel IgG Spin Purification Kit (Thermo, Waltham, MA, USA) [29,31,32,43,44]. The purification gel was transferred to a column coupled to a polypropylene conical tube and was then centrifuged. The supernatant was discarded, and the purification gel was resuspended in a mild purification buffer at physiological pH. The supernatant was discarded, and the purification gel was resuspended in the same purification buffer. A pooled serum sample from each group (HC or AD) was added to the gel, and the mixture was homogenized. The supernatant (purified IgG from AD patients—hereafter AD IgG; purified IgG from HC—hereafter HC IgG) was collected, sterilized, and stored at −80 °C for subsequent cell culture experiments. According to the manufacturer’s instructions, IgG concentration was determined using Coomassie Protein Assay Reagent (Pierce, Waltham, MA, USA). The purity of IgG, evaluated by SDS-PAGE, was above 95%. IgG isotypes (IgG1, IgG2, IgG3, and IgG4) were determined in purified IgG samples by ELISA (IgG Subclass Human ELISA Kit, ThermoFisher, USA) as per the manufacturer’s instructions. All technical steps were performed under sterile conditions, and endotoxin contamination was determined using the Pierce LAL Chromogenic Endotoxin Quantitation Kit (ThermoFisher, Waltham, MA, USA) to be undetectable levels (<0.01 EU/mL).

We used the Zenon Human IgG Labeling Kit (Invitrogen, Waltham, MA, USA) and followed the manufacturer’s instructions for IgG labeling. In brief, the Alexa-647 fluorophore attached to the monovalent affinity-purified Fab fragments directly recognizes the Fc portion of human IgG. Because this labeling method is immunoselective, it excludes the staining of any other proteins, including non-IgG antibodies, resulting in specific IgG staining. Thymocytes were incubated for 30 min with labeled IgG or, as controls, with Zenon labeling and blocking reagents without purified IgG or unlabeled IgG only. This method was standardized with 100 μg/mL of IgG, and the optimal concentration was determined by the culture experiments results. To validate this method, we confirmed that the previous incubation with the respective unlabeled purified IgG at the same concentration could completely block the staining provided by labeled purified IgG.

### 2.4. Cell Culture and Flow Cytometry

Cell cultures were performed as in previous studies [28,31], using purified IgG from adult AD patients and, as controls, purified IgG from adult HC, commercially used IgG for human intravenous administration that was obtained from thousands of healthy donors (IVIg—Endobulin Kiovig, Baxter, Lessines, Belgium) or the mock condition (absence of IgG). Briefly, we assessed thymocyte viability using a Neubauer chamber under an optical microscope (Laboroptik, Friedrichsdorf, Germany), and 1 × 10^6^ viable thymocytes were distributed to each well of a 48-well culture plate (CoStar, Glendale, CA, USA) with RPMI medium and 10% FCS (HyClone-III, Logan, UT, USA) with a total volume of 400 µL. Viable thymocytes were cultured in the absence (Mock control condition) or presence of 25, 50, or 100 μg/mL of IVIg, purified IgG from HC, or purified IgG from AD patients. All thymocyte cultures were performed in individual experiments. After incubating the culture plate for six days (a standardized period for observing lymphocyte maturation), thymocytes were transferred to test tubes for extracellular (phenotypic) staining. Thymocytes were fixed with formaldehyde and stained with mouse antihuman γδTCR-FITC, CD3-BV421, anti-CLA-PE, anti-α4-PECy5, anti-β7-BV605, or isotype control antibodies to identify γδT cells (CD3+γδTCR+) and evaluate the coexpression of α4 and β7 (α4β7+), or the expression of CLA in this population.

To evaluate thymocyte intracellular cytokines production, they were separately cultured in the same conditions (mock, IVIg, HC IgG, or AD IgG), but Brefeldin A (Sigma, Rehovot, Israel) was added to each well of the culture plate 12 h before cell staining [42,45]. This protocol was standardized using positive (Phorbol 12-myristate 13-acetate-PMA) and negative controls (mock condition), and due to the absence of polyclonal stimulation, we were able to maintain brefeldin A for 12 h without decreasing cell viability [27,28,44]. The culture conditions differ from phenotypic evaluation because we observed that brefeldin A could impair the detection of nonconstitutive surface molecules, including α4β7 and CLA, during the standardization period.

After the extracellular staining of γδT cells, samples were incubated with saponin. The supernatant was then removed, and cells were stained with mouse antihuman IFN-γ-APC, IL-9-PerCPCy5.5, IL-4-PE, IL-17-Alexa700, and IL-22A-PECy7, or an isotype control conjugated with the corresponding fluorochrome (BD Pharmingen, Franklin Lakes, NJ, USA).

For cell viability analysis, extracellular staining was performed as described above, and cells were stained with the Live/Dead (PE-Texas red) fluorescent reagent (ThermoFisher, Waltham, MA, USA). All antibodies, including the labeled IgG, were titrated until a concentration of 1 μg was determined to be optimal for specific staining. Cell gating was determined using the isotype control values or the fluorochrome minus 1 (FMO) setting to all parameters. All extracellular and intracellular analyses were performed on viable cells.

Using an LSRII Fortessa flow cytometer (BD Biosciences, Franklin Lakes, NJ, USA), 500,000 events per sample were acquired in the quadrant of lymphocytes. Adsorbed microspheres were used for compensation (CompBeads-BD Biosciences, Piscataway, NJ, USA). FlowJo software was used to analyze the data (Tree Star, Ashland, OR, USA).

## 3. RNA Extraction

To establish a single sample for each experimental condition, an equal number of viable thymocytes from 20 nonatopic neonatal patients were pooled together to have sufficient cells. RNA was extracted from cultured thymocytes from each condition using the miRCURY RNA Isolation kit (Exqon, Vedbæk, Denmark) as described in the manufacturer’s guidelines. The extracted RNA was eluted with RNase-free water and stored at −80 °C until use. Small RNAs were quantified using a Qubit 2.0 fluorometer (ThermoFisher, Waltham, MA, USA).

### 3.1. sRNA Construction and Sequencing

Sequence libraries were generated using the TruSeq Small R.N.A. sample preparation kit (Illumina, San Diego, CA, USA) as per the manufacturer’s instructions and a previously published protocol [44]. A total library pool of 4 nM was prepared using a MiSeq Reagent Kit v3 150 cycles followed by sequencing on a MiSeq system (Illumina, San Diego, CA, USA). The libraries were sequenced on a 150-SE run on the MiSeq with a 36 base single-end protocol [45]. After trimming the adapter sequences and sequence quality testing, each library’s raw data were aligned to the human reference genome (hg19), combined into an expression matrix, and processed with Strand NGS version 3.1 software (Strand Life Science Bangalore, Karnataka, India). Only miRNAs with more than ten copies were considered for subsequent analysis. miRNAs with a fold-change ≥ 2 were supposed to be differentially expressed. All sequence data described here are available in online repository Zenodo (https://doi.org/10.5281/zenodo.6470816, accessed on 14 June 2022).

### 3.2. Prediction, Gene Set Enrichment, and Functional Analysis of Target mRNA

We used online platform miRWalk v3 [46] to predict the target gene of miRNAs. The target genes were obtained by the intersection of three prediction software programs, TargetScan, miRDB, and miRTarBase, on the basis of the mirWALK v3 database. Functional and gene set enrichment analyses were carried out in miRWalk using the KEGG (KEGG Pathway Database, 2021) and REACTOME (Home—Reactome Pathway Database, 2021), and Gene Ontology (GO) terms to demonstrate the specific biological processes (BPs), cellular components (CCs), and molecular functions (MFs) associated with the selected miRNAs of the resulting gene sets.

## 4. Statistical Analysis

GraphPad Prism 8.0 was used for statistical analysis (GraphPad Software Inc., La Jolla, CA, USA). Data from in vitro studies were taken from 6 to 10 separate experiments with different thymus donors, as indicated in the figure legends. According to one-way ANOVA, differences were considered to be significant at *p* ≤ 0.05 (Kruskal–Wallis test, comparisons among three or more groups).

## 5. Results

### 5.1. AD IgG Can Modulate the Expression of α4β7 and CLA, and the Production of IL-17 and IL-22 by Human Neonatal Non-Atopic Thymic γδT Cells

**Figure 1:**
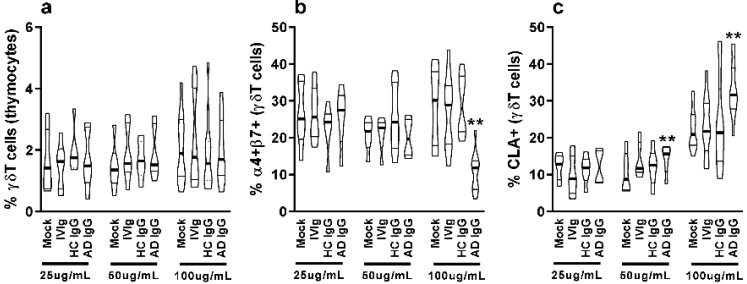
**Purified adult AD IgG’s effect on the homing phenotype of infant nonatopic intra-thymic γδT cells.** Thymocytes from children under 7 days old (*n* = 12) were evaluated after 6 days in culture in RPMI medium supplemented with FCS in the absence (mock) or presence of 25, 50, or 100 µg/mL of commercially used purified IgG (IVIg), or IgG purified from health controls (HC IgG) or IgG purified from adult AD patients (AD IgG). Flow cytometry was used to determine the frequency of γδT (CD3+γδTCR+) cells (**a**) or the expression of α4+β7+ (**b**) and CLA (**c**) in γδT (CD3+γδTCR+). Violin plots depict the distribution of values from lowest to highest, with thick lines representing quartiles, and bold lines representing the mean. ** *p* ≤ 0.05 when compared to Mock, IVIg, and HC IgG conditions.

**Figure 2:**
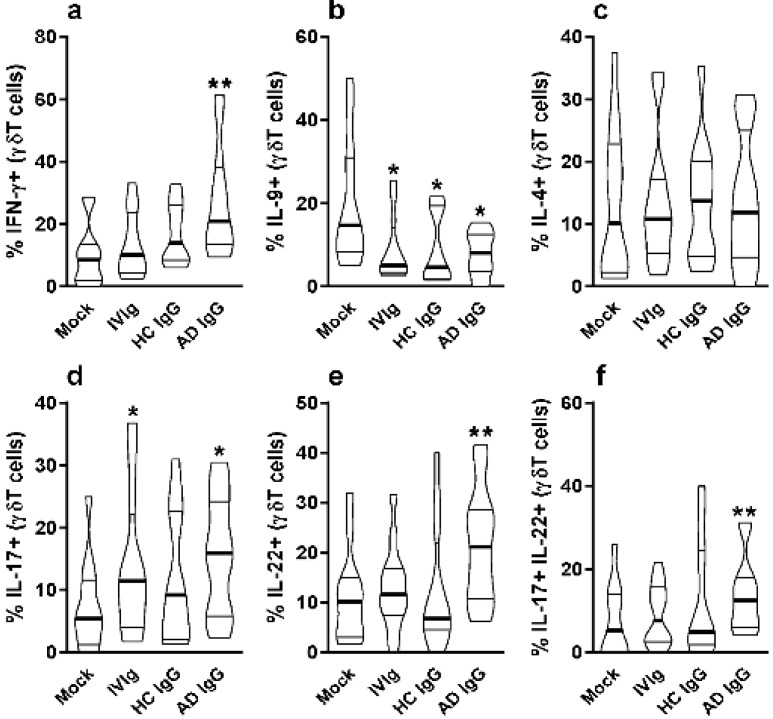
**Effect of purified adult AD IgG on the cytokine production of infant nonatopic intra-thymic γδT cells.** Thymocytes from children under 7 days old (*n* = 12) were evaluated after 6 days in culture in RPMI medium supplemented with FCS in the absence (mock) or presence of 100 µg/mL of commercially used purified IgG (IVIg) or IgG purified from health controls (HC IgG) or IgG purified from adult AD patients (AD IgG). Flow cytometry was used to determine the intracellular production of IFN-γ (**a**), IL-9 (**b**), IL-4 (**c**), IL-17 (**d**), IL-22 (**e**) or the coproduction of IL-17 and IL-22 (**f**) were evaluated in γδT (CD3+γδTCR+) cells. Violin plots shows the distribution of values from lowest to highest, with thick lines representing quartiles and bold lines representing the mean. * *p* ≤ 0.05 when compared to the mock condition. ** *p* ≤ 0.05 when compared to the Mock, IVIg, and HC IgG conditions.

Nonatopic neonatal thymocytes were cultured and compared to mock or IVIg to investigate the in vitro effect of AD IgG. We first investigated the influence of three different concentrations of IgG on the frequency of γδT cells as well as the expression of mucosal-related (α4β7) and skin-related (CLA) homing molecules in these cells. γδT cells constituted nearly 2% of all thymocytes, and the concentration of IgG had no effect on the observed percentage (Figure 1a); however, AD IgG at higher concentrations could inhibit the expression of the α4β7 molecule, and induce the expression of CLA in γδT cells (Figure 1b,c). CLA induction in γδT cells was also seen in response to an intermediate dose of AD IgG (Figure 1c). As a result of these observations, we decided to perform further experiments using only the higher dose (100 ug/mL). Next, we assessed the cytokine production profile of thymocytes in response to AD IgG and control conditions. Results reveal that AD IgG could augment the production of IFN-γ by γδT cells when compared to controls (Figure 2a).

On the other hand, IL-9 production by γδT cells was inhibited by AD IgG, HC IgG, and IVIg compared to the mock condition (Figure 2b). There was no effect in γδT cell IL-4 production in any culture condition (Figure 2c). However, only AD IgG and IVIg were able to induce the IL-17-producing γδT cells when compared to the mock condition (Figure 2d), and only AD IgG was able to induce IL-22-producing γδT cells in comparison to the controls (Figure 2e). Due to the possibility of IL-17 and IL-22 coproduction by γδT cells, we also evaluated the frequency of double-positive (IL-17+IL-22+) γδT cells, and found that only AD IgG could induce a higher frequency of these cells compared to control conditions (Figure 2f).

### 5.2. AD IgG Can Directly Interact with Nonatopic Neonatal Thymic γδT Cells Membrane and Regulate the Induction of Apoptosis

**Figure 3:**
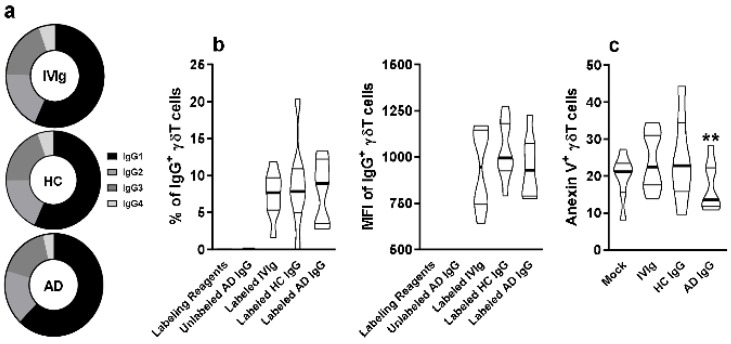
**Evaluation of IgG subclass frequency and their direct interaction with thymic γδT cells.** (**a**) Frequency of IgG1, IgG2, IgG3, and IgG4 isotypes in IVIg and purified AD IgG was evaluated. Thymocytes from children under 7 days old (*n* = 12) were incubated for 30 min with labeling kit reagents (Without IgG), unlabeled AD IgG, labeled IVIg, labeled HC IgG, or labeled AD IgG. (**b**) Frequency and (**c**) intensity of IgG staining (IgG+) or (**c**) frequency of Anexin V staining (Anexin V+) on thymic γδT (CD3+γδTCR+) cells. Pie charts represent the frequency of each IgG isotype within the total amount of detected IgG. Violin plots represents values distribution from minimum to maximum, thick lines represent quartiles, and bold lines represent the mean. ** *p* ≤ 0.05 when compared to the Mock, IVIg, and HC IgG conditions.

To investigate the possibility of direct interaction of IgG molecules with thymic γδT cells membrane, we first evaluated the frequency of IgG subclasses IgG1, IgG2, IgG3, and IgG4 on IVIg, HC IgG, and AD IgG. No differences could be observed among the pooled sera from each group (Figure 3a).

Next, we stained IVIg, HC IgG, and AD IgG with a fluorophore, and measured the frequency and intensity of detection on the thymic γδT cell membrane. Our results indicate that all stained IgG formulations could interact at a similar frequency and intensity with thymic γδT cells (Figure 3b). However, only AD IgG could reduce the frequency of Annexin V+ γδT cells, indicating a reduction in phosphatidylserine expression, hence reducing early apoptosis induction compared to IVIg, HC IgG, and mock conditions (Figure 3c).

### 5.3. Identification of Differentially Expressed miRNAs and Their Target Genes

**Figure 4:**
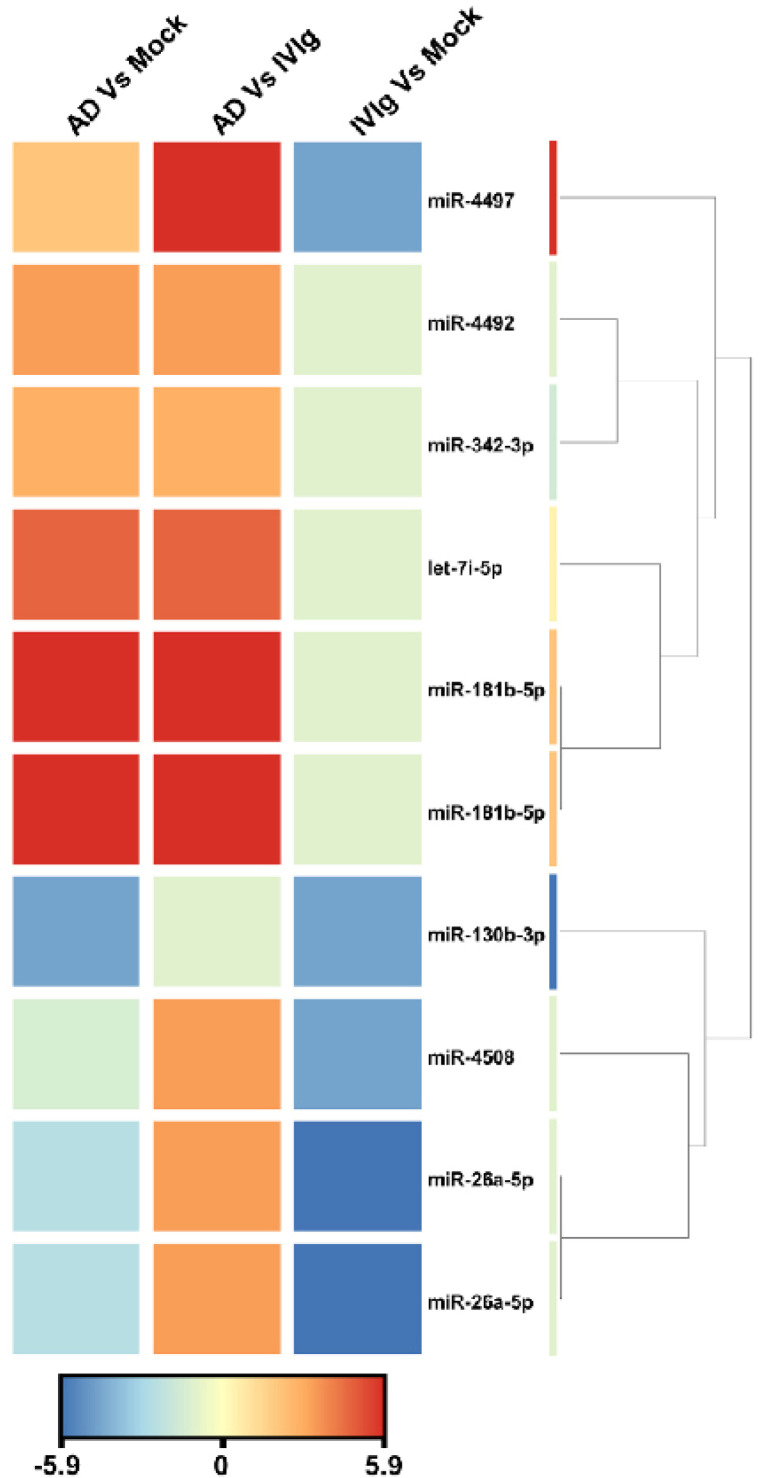
**Unsupervised hierarchical clustering of significantly dysregulated miRNAs and samples from the Illumina deep sequencing data.** The heat map contains 10 miRNAs differentially expressed in AD vs. Mock control, AD vs. IVIg, and IVIg vs. mock control. The miRNA clustering tree is displayed on the right. The color scale indicates the relative expression levels of miRNA across all samples. Red indicates that the expression levels are higher than the mean, whereas blue indicates that the expression levels are lower than the mean. Each row represents one mature miRNA, and each column represents one sample. AD: thymocytes stimulated by IgG from patients with atopic dermatitis; IVIg: thymocytes stimulated by therapeutic intravenous IgG.

To identify miRNA expression signatures associated with thymocytes, we analyzed active miRNA expression profiles using the Illumina small RNA sequencing approach in three culture conditions: the absence of IgG (Mock), the therapeutic IgG formulation (IVIg), and the experimental condition with AD IgG. When we compared AD IgG with IVIg, sequencing analysis revealed nine overexpressed miRNAs that satisfied the Bonferroni-corrected value criterion (*p* ≤ 0.05) and the fold-change criterion (FC ≥ 2), showing considerable variation between AD IgG and IVIg controls (Figure 4). When AD IgG was compared to a mock condition, nine miRNAs were significantly dysregulated, with six and three miRNAs being over- and underexpressed, respectively (Figure 4). Lastly, the deep sequencing approach identified five miRNAs underexpressed in response to IVIg compared to the mock condition (Figure 4). Hierarchical clustering based on the significantly dysregulated miRNAs showed a clear separation between AD IgG and control conditions. hsa-miR-4497 and hsa-miR-181b-5p (fold changes, 9.5 and 6.17, respectively) were the most upregulated miRNAs, and hsa-miR-130b-3p was the most downregulated in response to AD IgG when compared to the mock condition (fold change, 4.5).

**Figure 5:**
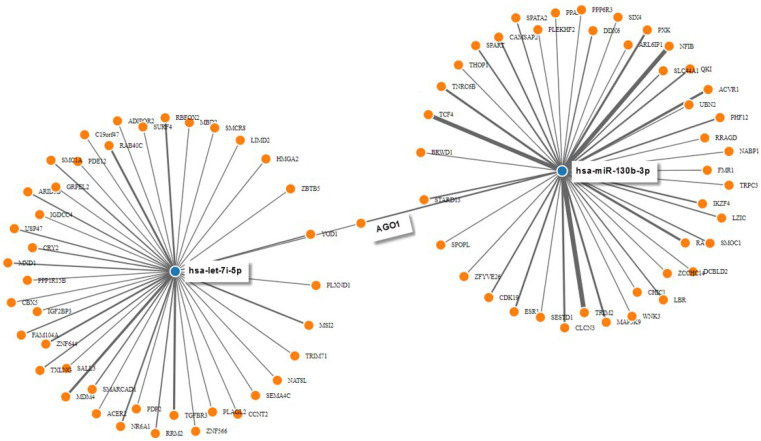
**Predicted interaction between miRNAs and target genes.** MiRNA-target gene interactions were generated manually by intersecting individual networks generated from miRWalk target gene prediction data. Only hsa-miR-130b-3p and hsa-let-7i-5p are displayed for clarity. Blue dots represent miRNAs that interact with their target genes, which are represented by orange dots.

The targetome prediction of seven nonredundant dysregulated miRNAs (hsa-miR-181b-5p, 26a-5p, 4492, let-7i-5p, 4497, 342-3p, and miR-4508) between AD IgG and mock condition revealed 122 putative targets from miRWalk. As shown in Figure 5, the miRNAs with the most experimentally validated targets (over 40 each) were hsa-miR-130b-3p and Hsa-let-7i-5p. Figure 5 also shows that hsa-let-7i-5p and hsa-miR-130b-3p synergistically target AGO1. This protein resides in intracellular structures known as Processing-bodies (P-bodies). This discrete area of the cells is believed to govern the cellular mRNA turnover [47].

### 5.4. Functional and Pathway Enrichment Analysis

Reactome, KEGG pathway, GO annotations, and enrichment analysis of the validated 122 target genes were computed by the miRWalk v3.0 online tool. Sixteen Reactome pathways were significantly enriched (Table S1), of which the transcriptional regulation of white adipocyte differentiation (R-HSA-381340) and transcriptional regulation by MECP2 (R-HSA-8986944) were the most enriched pathways (Table S1). Regarding the GO BPs, the upregulated genes were mainly related to the TGF-β receptor signaling pathway and unfavorable translation, transcription, and cell differentiation regulation. GO CCs mainly included the p-body, and GO MFs were mainly enriched in RNA binding and nuclear receptor activity. Upregulated genes were significantly and uniquely involved in transcriptional misregulation in cancer regarding the KEGG pathways.

## 6. Discussion

To evaluate the effect of AD IgG antibodies on cytokine production by thymic γδT cells using a well-established method [48,49,50,51,52,53], we collected human thymus from nonatopic infants who were chosen because their mothers had no allergy history. The culture protocol had previously been developed as an in vitro model to investigate the modulatory effect of purified IgG on murine and human thymic and peripheral cells [24,25,26,28,30,32,40,54,55,56].

In the present study, AD IgG could downregulate the expression of the α4β7 molecules. Integrin α4β7 is a heterodimeric transmembrane glycoprotein expressed as a homing receptor in lymphocyte membranes that mediates trafficking to the gut-associated lymphoid tissue via the interaction with mucosal addressin cell adhesion molecule-1 (MAdCAM-1), which is predominantly expressed in the intestinal mucosa [57] but also in the lungs [58]. Therefore, the low expression of the α4β7 heterodimer by γδT cells in response to AD IgG suggested that these cells are not prone to migrating to mucosal sites. Otherwise, AD IgG could upregulate the expression of CLA. This molecule is a primary skin-homing molecule expressed by T cells, and a peripheral biomarker of AD [59]. Previous studies showed that adults with AD have a higher frequencies of IL-22-producing TCD4 cells with high CLA expression [17]. As a result, the overexpression of CLA in γδT cells suggests that AD IgG may modulate intrathymic γδT cells maturation, favoring its migration to the skin.

We investigated the cytokine production profile of γδT cells and we found that AD IgG might increase the production of IFN-γ, IL-17, and IL-22 by thymic γδT cells. Higher levels of IFN-γ in AD serum had been described in adult AD [60], and they appeared to occur in the absence of substantial IFN-γ production by skin-derived TCD4 cells [61]. Other investigators, on the other hand, observed that skin-derived TCD4 cells produce lower levels of IFN-γ when compared to psoriasis-skin-derived TCD4 cells [62], and that pediatric or adult AD skin can be characterized by decreased IFN-γ expression [63]. Our observations reveal that γδT cells may be implicated in the IFN-γ production, opening up a new avenue for understanding IFN-γ systemic output in AD patients.

IL-17 is a major cytokine of AD that induces inflammatory proteins in the pathogenesis of atopic dermatitis [64]. IL-17 production can result in eosinophil- and neutrophil-mediated inflammation, whereas low IL-17 levels are linked to skin infection susceptibility [65,66]. A previous study found that IL-17 production in AD lesions and its levels in sera are related to the severity of AD disorder [67]. Another study found that IL-17 upregulation is a feature of AD initiation in children’s skin when compared to adult AD [68].

Our results also show that neonatal thymic γδT cells produce more IL-17 output in response to AD IgG. The induction of IL-17 production mediated by AD IgG was described in the literature in a similar thymic in vitro model, but with a focus on thymic iNKT, TCD4, and TCD8 cells [28,31]. IgG from allergic (atopic but not AD) patients also induced in vitro IL-17 production, and this production was induced in TCD8 [53] and γδT [32] cells.

As previously stated, adults with AD had increased frequencies of IL-22 production by TCD4 cells [17], and the role of IL-22 in AD pathogenesis could be demonstrated in a clinical trial using a neutralizing anti-IL-22 antibody that could induce progressive and sustained clinical improvements in adult AD patients [19]. This was rendered possible by IL-22’s important role in the maintenance of skin homeostasis [15]. Furthermore, AD IgG could induce γδT cells IL-22-production. The role of γδT cells as a source of IL-22 in AD pathogenesis is unknown, but the induction of IL-17- and IL-22-producing γδT cells is comparable [69] and may involve some molecules that are not described as γδT cell modulators. The same study found a low frequency of peripheral IL-17/IL-22 coproduction by γδT cells (around 3%), whereas another study showed a higher frequency (near 20%) of coproduction by TCD4 cells [14]. The acquisition of IL-17/IL-22 coproduction by thymic γδT cells is unknown; we also evaluated this parameter in thymic γδT cells. Our results show that about 10% of the total γδT cells can coproduce IL-17 and IL-22 in response to AD IgG, which is similar to previous studies. However, in our study, this frequency represents nearly 50% of the γδT cells that produce IL-17 or IL-22. This latter observation implies that the coproduction of IL-17 and IL-22 γδT cells, mainly in primary organs, requires further investigation and may differ from peripheral observations.

Although the modulatory effect of AD IgG was demonstrated in terms of phenotypic and functional features, the potential mechanisms that could directly link IgG to γδT cell regulation were lacking in our study and the literature. To develop an elucidative approach to these mechanisms, we evaluated whether IVIg and AD IgG could interact with the membrane of γδT cells. Our results reveal that IgG molecules could directly interact with the thymic γδT cell membrane, which was previously unknown in the literature. These interactions may be mediated by the idiotypic recognition of membrane receptors (including clonal γδTCR) expressed by immature lymphocytes [70,71,72,73]. Although only a small proportion of γδT cells can express IgG receptors in their membranes (CD16/FcγR3A), they are functionally differentiated by CTL activity [74]. The staining protocol used in our study was based on fluorophores attached to the monovalent affinity-purified Fab fragments directed against the Fc portion of IgG primary antibodies [75]. Therefore, the staining method hinders CD16 interactions by occupying the Fc portion of the IgG molecule, even allowing for simultaneous CD16 identification in complex cytometry staining panels as described in the literature [76].

Although the characterization of γδTCRs ligands is relatively new [77] and still being debated [5], γδTCRs signaling modulates three major aspects of γδT cell biology: (i) homing properties [78], (ii) IL-17 and IFN-γ production [79], and (iii) apoptosis [80]. Our findings show that AD IgG could directly interact with thymic γδT cells and modulate the three aforementioned biological aspects of γδT cells. These findings strongly suggest that the AD IgG effect is related to γδTCR signaling.

To investigate the effect of AD IgG and its role in the development of human AD, we decided to assess if there was an epigenetic signature that allowed for us to observe a relationship between the in vitro observations and molecular characteristics described in AD patients. Results reveal nine significantly deregulated miRNAs in AD IgG-induced thymocytes vs. mock (Table S1), with many of them predicted to target genes involved in metabolic pathways and transcriptional dysregulation in cancer. The forced expression of hsa-miR-4497 suppresses proliferation and colony formation while inducing the apoptosis of laryngeal squamous cell carcinoma cells by repressing antiapoptotic Bcl-2 proteins [81]. In addition to its roles in tumorigenesis, hsa-miR-4497 mediates oxidative stress and inflammatory injury in keratinocytes by regulating NF-κB expression [82]. Notably, oxidative stress was linked to the pathogenesis of AD [83,84]. Thus, it is possible that hsa-miR-4497 overexpression in AD skin may promote both oxidative damage and NF-kB activation. According to the literature, the overexpression of miR-181b-5p induces B- and T-cell differentiation when ectopically expressed in hematopoietic stem or progenitor cells [38,85]. Thus, the abundant expression of miR-181b-5p in thymocytes suggests that this miRNA may influence the function of B or T cells in AD. The other dysregulated has-miR-4492, 26a-5p, 4492, 4497, 342-3p, and 130b-3p have not been previously described in the context of AD, and our results suggest that these miRNAs may play a role in AD pathogenesis. Consistent with previous data published by Sonkoly et al. [86], who the showed elevated expression of has-let-7i-5p in the skin lesions of AD patients, this miRNA was significantly upregulated in thymocytes derived from the AD IgG condition compared to the mock condition. The expression of has-miR-4508 did not show any significant differences between AD IgG and mock, indicating that it is likely less relevant in the pathogenic mechanism of AD.

In order to search for pathways that are relevant to AD, we searched the MirWlak-database for target genes for the seven dysregulated miRNAs between AD IgG and the mock condition, and found relevant Reactome pathways, namely, transcriptional regulation of white adipocyte differentiation. This pathway was found from the target genes for thashsa-miR-130hasp, hsa-miR-181bhas, and hsa-miR-26a-5p. A recent study by Yew et al. [87] investigated the causal relationship between AD and obesity by performing Mendelian randomization analysis using data extracted from GWASs of body mass index and AD. Their results indicated a causal role of adiposity in the development of AD, and suggested that obesity is a risk factor and a facilitator for the development of AD. Although the mechanism that links obesity and AD is unknown, available data suggest that a chronic inflammatory state caused by free fatty acids, adipokines, and adipocytokines from adipocytes and other local cells can mediate the increased risk of AD in obese patients [88]. Our findings are the first to show that changes induced by IgG from AD patients also affect miRNA expression in thymocytes, thus influencing their metabolism in AD. The Reactome pathway also revealed that transcriptional regulation by methyl-CpG-binding protein 2 (MECP2) was the second most significant pathway enriched by target genes. A recent study by He et al. [89] showed a novel role for MeCP2 in skin fibrosis in systemic sclerosis (scleroderma, SSc). Skin fibrosis is a principal feature in AD characterized by elevated IL-13 and thymic stromal lymphopoietin in skin lesions. Thus, we speculated that the dysregulated miRNAs and their target genes might be involved in AD’s progression via interference in the MeCP2 pathway. However, further investigation is needed to clarify this speculation.

In this study, NF1B was one of the outstanding target genes that controlled has-miR- 130 on the basis of the calculation of the network. Available data indicate that the deregulation of this gene is directly associated with melanocyte function [90], and that the loss of this gene induces the apoptosis of adjacent hair germs that take up melanin. Although there is no direct evidence that NF1B is involved in AD, we speculated that the dysregulation of this gene might be linked to the development of AD.

In conclusion, our cellular and molecular observations suggest that IgG from adult AD patients can modulate the nonatopic neonatal thymic maturation of γδT cells, allowing for them to acquire functional properties that contribute to the development of AD. The mechanism underlying this observation is unknown, but it may involve direct idiotypic interactions and a complex epigenetic regulation that need to be elucidated.

## Supplementary Materials

The following supporting information can be downloaded at: https://www.mdpi.com/article/10.3390/ijms23126872/s1.

## References

[1] Ferreira L.M. (2013). Gammadelta T cells: Innately adaptive immune cells?. Int. Rev. Immunol..

[2] Brenner M.B., McLean J., Dialynas D.P., Strominger J.L., Smith J.A., Owen F.L., Seidman J.G., Ip S., Rosen F., Krangel M.S. (1986). Identification of a putative second T-cell receptor. Nature.

[3] Lanier L.L., Serafini A.T., Ruitenberg J.J., Cwirla S., Federspiel N.A., Phillips J.H., Allison J.P., Weiss A. (1987). The gamma T-cell antigen receptor. J. Clin. Immunol..

[4] Satyanarayana K., Hata S., Devlin P., Roncarolo M.G., De Vries J.E., Spits H., Strominger J.L., Krangel M.S. (1988). Genomic organization of the human T-cell antigen-receptor alpha/delta locus. Proc. Natl. Acad. Sci. USA.

[5] de Sousa T.R., Victor J.R. (2020). Natural Self-Ligand Gamma Delta T Cell Receptors (γδTCRs) Insight: The Potential of Induced IgG. Vaccines.

[6] Pang D.J., Neves J.F., Sumaria N., Pennington D.J. (2012). Understanding the complexity of γδ T-cell subsets in mouse and human. Immunology.

[7] de Oliveira Henriques M.D., Penido C. (2012). γδ T Lymphocytes Coordinate Eosinophil Influx during Allergic Responses. Front. Pharmacol..

[8] Huang Y., Aydintug M.K., Loomis J., Macleod M.K., McKee A.S., Kirchenbaum G., Jakubzick C.V., Kedl R.M., Sun D., Jacobelli J. (2013). Antigen-specific regulation of IgE antibodies by non-antigen-specific γδ T cells. J. Immunol..

[9] Cairo C., Arabito E., Landi F., Casati A., Brunetti E., Mancino G., Galli E. (2005). Analysis of circulating gammadelta T cells in children affected by IgE-associated and non-IgE-associated allergic atopic eczema/dermatitis syndrome. Clin. Exp. Immunol..

[10] Jee M.H., Mraz V., Geisler C., Bonefeld C.M. (2020). γδ T cells and inflammatory skin diseases. Immunol. Rev..

[11] Castillo-González R., Cibrian D., Sánchez-Madrid F. (2021). Dissecting the complexity of γδ T-cell subsets in skin homeostasis, inflammation, and malignancy. J. Allergy Clin. Immunol..

[12] Spidale N.A., Malhotra N., Frascoli M., Sylvia K., Miu B., Freeman C., Stadinski B.D., Huseby E., Kang J. (2020). Neonatal-derived IL-17 producing dermal γδ T cells are required to prevent spontaneous atopic dermatitis. eLife.

[13] Ness-Schwickerath K.J., Morita C.T. (2011). Regulation and function of IL-17A- and IL-22-producing γδ T cells. Cell Mol. Life Sci..

[14] Duhen T., Geiger R., Jarrossay D., Lanzavecchia A., Sallusto F. (2009). Production of interleukin 22 but not interleukin 17 by a subset of human skin-homing memory T cells. Nat. Immunol..

[15] Fukaya T., Fukui T., Uto T., Takagi H., Nasu J., Miyanaga N., Arimura K., Nakamura T., Koseki H., Choijookhuu N. (2018). Pivotal Role of IL-22 Binding Protein in the Epithelial Autoregulation of Interleukin-22 Signaling in the Control of Skin Inflammation. Front. Immunol..

[16] Czarnowicki T., Gonzalez J., Shemer A., Malajian D., Xu H., Zheng X., Khattri S., Gilleaudeau P., Sullivan-Whalen M., Suárez-Fariñas M. (2015). Severe atopic dermatitis is characterized by selective expansion of circulating TH2/TC2 and TH22/TC22, but not TH17/TC17, cells within the skin-homing T-cell population. J. Allergy Clin. Immunol..

[17] Czarnowicki T., Esaki H., Gonzalez J., Malajian D., Shemer A., Noda S., Talasila S., Berry A., Gray J., Becker L. (2015). Early pediatric atopic dermatitis shows only a cutaneous lymphocyte antigen (CLA)(+) TH2/TH1 cell imbalance, whereas adults acquire CLA(+) TH22/TC22 cell subsets. J. Allergy Clin. Immunol..

[18] Hijnen D., Knol E.F., Gent Y.Y., Giovannone B., Beijn S.J., Kupper T.S., Bruijnzeel-Koomen C.A., Clark R.A. (2013). CD8(+) T cells in the lesional skin of atopic dermatitis and psoriasis patients are an important source of IFN-γ, IL-13, IL-17, and IL-22. J. Investig. Dermatol..

[19] Guttman-Yassky E., Brunner P.M., Neumann A.U., Khattri S., Pavel A.B., Malik K., Singer G.K., Baum D., Gilleaudeau P., Sullivan-Whalen M. (2018). Efficacy and safety of fezakinumab (an IL-22 monoclonal antibody) in adults with moderate-to-severe atopic dermatitis inadequately controlled by conventional treatments: A randomized, double-blind, phase 2a trial. J. Am. Acad. Dermatol..

[20] Victor J.R. (2020). Do different IgG repertoires play a role in B- and T-cell functional modulation during ontogeny? The “hooks without bait” theory. Immunol. Cell Biol..

[21] Victor J.R. (2017). Allergen-specific IgG as a mediator of allergy inhibition: Lessons from mother to child. Hum. Vaccin. Immunother..

[22] Victor J.R. (2014). Influence of maternal immunization with allergens on the thymic maturation of lymphocytes with regulatory potential in children: A broad field for further exploration. J. Immunol. Res..

[23] Victor J., Fusaro A., Duarte A., Sato M. (2003). Preconception maternal immunization to dust mite inhibits the type I hypersensitivity response of offspring. J. Allergy Clin. Immunol..

[24] Victor J.R., Muniz B.P., Fusaro A.E., de Brito C.A., Taniguchi E.F., Duarte A.J.S., Sato M.N. (2010). Maternal immunization with ovalbumin prevents neonatal allergy development and up-regulates inhibitory receptor Fc gamma RIIB expression on B cells. BMC Immunol..

[25] de Oliveira M.G., de Mendonça Oliveira L., de Lima Lira A.A., Sgnotto F.d.R., da Silva Duarte A.J., Sato M.N., Victor J.R. (2017). Preconception allergen sensitization can induce B10 cells in offspring: A potential main role for maternal IgG. Allergy Asthma Clin. Immunol..

[26] Lira A.A.L., de-Oliveira M.G., Inoue A.H.S., Beltrame G.R., Duarte A.J.D.S., Victor J.R. (2018). Preconceptional allergen immunization can induce offspring IL-17 secreting B cells (B17): Do they share similarities with regulatory B10 cells?. Allergol. Immunopathol..

[27] Sgnotto F.D.R., Oliveira M.G., Lira A.A.L., Bento-de-Souza L., Duarte A.J.D.S., Victor J.R. (2017). Low doses of IgG from atopic individuals can modulate in vitro IFN-γ production by human intra-thymic TCD4 and TCD8 cells: An IVIg comparative approach. Hum. Vaccin. Immunother..

[28] Sgnotto F.D.R., de Oliveira M.G., Lira A.A.L., Inoue A.H.S., Titz T.O., Orfali R.L., Bento-de-Souza L., Sato M.N., Aoki V., Duarte A.J.S. (2018). IgG from atopic dermatitis patients induces IL-17 and IL-10 production in infant intrathymic TCD4 and TCD8 cells. Int. J. Dermatol..

[29] da Ressureição Sgnotto F., Souza Santos L., Rodrigues de Sousa T., Feitosa de Lima J., da Silva Oliveira L.M., Saeed Sanabani S., da Silva Duarte A.J., Russo Victor J. (2019). IgG From HIV-1-Exposed Seronegative and HIV-1-Infected Subjects Differently Modulates IFN-γ Production by Thymic T and B Cells. J. Acquir. Immune. Defic. Syndr..

[30] de Sousa T.R., da Ressureição Sgnotto F., Fagundes B.O., da Silva Duarte A.J., Victor J.R. (2021). Non-atopic Neonatal Thymic Innate Lymphoid Cell Subsets (ILC1, ILC2, and ILC3) Identification and the Modulatory Effect of IgG from Dermatophagoides Pteronyssinus (Derp)-Atopic Individuals. Front. Allergy.

[31] Santos L.S., Sgnotto F.D.R., Sousa T.R., Orfali R.L., Aoki V., Duarte A.J.D.S., Victor J.R. (2019). IgG from atopic dermatitis patients induces non-atopic infant thymic invariant natural killer T (iNKT) cells to produce IL-4, IL-17, and IL-10. Int. J. Dermatol..

[32] de Oliveira M.G., de Lima Lira A.A., da Ressureição Sgnotto F., Inoue A.H.S., Santos L.S., Nakamatsu B.Y., Duarte A.J.D.S., Leite-de-Moraes M., Victor J.R. (2019). Maternal IgG impairs the maturation of offspring intrathymic IL-17-producing γδT cells: Implications for murine and human allergies. Clin. Exp. Allergy.

[33] Santos L.S., Sgnotto F.D.R., Inoue A.H.S., Padreca A.F., Menghini R.P., Duarte A.J.D.S., Victor J.R. (2019). IgG from Non-atopic Individuals Induces In Vitro IFN-γ and IL-10 Production by Human Intra-thymic γδT Cells: A Comparison with Atopic IgG and IVIg. Arch. Immunol. Ther. Exp..

[34] Ribot J.C., de Barros A., Pang D.J., Neves J.F., Peperzak V., Roberts S.J., Girardi M., Borst J., Hayday A.C., Pennington D.J. (2009). CD27 is a thymic determinant of the balance between interferon-gamma- and interleukin 17-producing gammadelta T cell subsets. Nat. Immunol..

[35] Ambros V. (2004). The functions of animal microRNAs. Nature.

[36] Baltimore D., Boldin M.P., O’Connell R.M., Rao D.S., Taganov K.D. (2008). MicroRNAs: New regulators of immune cell development and function. Nat. Immunol..

[37] Wells A.C., Daniels K.A., Angelou C.C., Fagerberg E., Burnside A.S., Markstein M., Alfandari D., Welsh R.M., Pobezinskaya E.L., Pobezinsky L.A. (2017). Modulation of let-7 miRNAs controls the differentiation of effector CD8 T cells. eLife.

[38] Chen C.Z., Li L., Lodish H.F., Bartel D.P. (2004). MicroRNAs modulate hematopoietic lineage differentiation. Science.

[39] Yu X., Wang M., Li L., Zhang L., Chan M.T.V., Wu W.K.K. (2020). MicroRNAs in atopic dermatitis: A systematic review. J. Cell Mol. Med..

[40] de-Sousa T.R., Pessôa R., Nascimento A., Fagundes B.O., Sgnotto F.D.R., Duarte A.J.D.S., Sanabani S.S., Victor J.R. (2021). Preconceptional Immunization Can Modulate Offspring Intrathymic IL-17-Producing γδT Cells with Epigenetic Implications Mediated by microRNAs. Int. J. Mol. Sci..

[41] Hanifin J.M., Thurston M., Omoto M., Cherill R., Tofte S.J., Graeber M. (2001). The eczema area and severity index (EASI): Assessment of reliability in atopic dermatitis. EASI Evaluator Group. Exp. Dermatol..

[42] Bento-de-Souza L., Victor J.R., Bento-de-Souza L.C., Arrais-Santos M., Rangel-Santos A.C., Pereira-Costa É., Raniero-Fernandes E., Seixas-Duarte M.I., Oliveira-Filho J.B., Duarte A.J.S. (2016). Constitutive expression of genes encoding notch receptors and ligands in developing lymphocytes, nTreg cells and dendritic cells in the human thymus. Results Immunol..

[43] Inoue A.H.S., Lira A.A.L., de-Oliveira M.G., de Sousa T.R., Sgnotto F.D.R., Duarte A.J.D.S., Victor J.R. (2020). The Potential of IgG to Induce Murine and Human Thymic Maturation of IL-10+ B Cells (B10) Revealed in a Pilot Study. Cells.

[44] de Souza D.R.V., Pessoa R., Nascimento A., Nukui Y., Pereira J., Casseb J., de Oliveira A.C.P., da Silva Duarte A.J., Clissa P.B., Sanabani S.S. (2020). Small RNA profiles of HTLV-1 asymptomatic carriers with monoclonal and polyclonal rearrangement of the T-cell antigen receptor gamma-chain using massively parallel sequencing: A pilot study. Oncol. Lett..

[45] Nascimento A., de Souza D.R.V., Pessoa R., Pietrobon A.J., Nukui Y., Pereira J., Casseb J., de Oliveira A.C.P., Loureiro P., da Silva Duarte A.J. (2021). Global expression of noncoding RNome reveals dysregulation of small RNAs in patients with HTLV-1-associated adult T-cell leukemia: A pilot study. Infect. Agents Cancer.

[46] Sticht C., De La Torre C., Parveen A., Gretz N. (2018). miRWalk: An online resource for prediction of microRNA binding sites. PLoS ONE.

[47] Sheth U., Parker R. (2003). Decapping and decay of messenger RNA occur in cytoplasmic processing bodies. Science.

[48] Mara-Koosham G., Hutt J.A., Lyons C.R., Wu T.H. (2011). Antibodies contribute to effective vaccination against respiratory infection by type A Francisella tularensis strains. Infect. Immun..

[49] Mahan A.E., Tedesco J., Dionne K., Baruah K., Cheng H.D., De Jager P.L., Barouch D.H., Suscovich T., Ackerman M., Crispin M. (2015). A method for high-throughput, sensitive analysis of IgG Fc and Fab glycosylation by capillary electrophoresis. J. Immunol. Methods.

[50] Dugast A.S., Chan Y., Hoffner M., Licht A., Nkolola J., Li H., Streeck H., Suscovich T.J., Ghebremichael M., Ackerman M.E. (2014). Lack of protection following passive transfer of polyclonal highly functional low-dose non-neutralizing antibodies. PLoS ONE.

[51] Ankeny D.P., Guan Z., Popovich P.G. (2009). B cells produce pathogenic antibodies and impair recovery after spinal cord injury in mice. J. Clin. Investig..

[52] Rigato P.O., Maciel M., Goldoni A.L., Piubelli O.G., Orii N.M., Marques E.T., August J.T., Duarte A.J., Sato M.N. (2012). Maternal LAMP/p55gagHIV-1 DNA immunization induces in utero priming and a long-lasting immune response in vaccinated neonates. PLoS ONE.

[53] de Sousa T.R., da Ressureição Sgnotto F., Oliveira Fagundes B., Souza Santos L., da Silva Duarte A.J., Victor J.R. (2021). IgG from atopic individuals can mediate non-atopic infant thymic and adult peripheral CD8. Eur. Ann. Allergy Clin. Immunol..

[54] de Oliveira M.G., da Ressureição Sgnotto F., de Sousa T.R., Fagundes B.O., Duarte A.J.D.S., Victor J.R. (2020). Preconceptional immunization with an allergen inhibits offspring thymic Th17 cells maturation without influence on Th1 and Th2 cells. Eur. Cytokine Netw..

[55] Futata E., de Brito C., Victor J., Fusaro A., Oliveira C., Maciel M., Duarte A., Sato M. (2006). Long-term anergy in orally tolerized mice is linked to decreased B7.2 expression on B cells. Immunobiology.

[56] de Oliveira M.G., Lira A.A.L., Sgnotto F.D.R., Inoue A.H.S., Beltrame G.R., da Silva D., Menghini R.P., Duarte A.J.D.S., Victor J.R. (2018). Maternal immunization downregulates offspring TCD4 regulatory cells (Tregs) thymic maturation without implications for allergy inhibition. Scand. J. Immunol..

[57] Briskin M., Winsor-Hines D., Shyjan A., Cochran N., Bloom S., Wilson J., McEvoy L.M., Butcher E.C., Kassam N., Mackay C.R. (1997). Human mucosal addressin cell adhesion molecule-1 is preferentially expressed in intestinal tract and associated lymphoid tissue. Am. J. Pathol..

[58] Kaminuma O., Saeki M., Nishimura T., Kitamura N., Watanabe N., Hiroi T., Mori A. (2017). Differential Contribution of Adhesion Molecules to Th1 and Th2 Cell-Mediated Lung and Bowel Inflammation. Biol. Pharm. Bull..

[59] Czarnowicki T., Santamaria-Babí L.F., Guttman-Yassky E. (2017). Circulating CLA^+^ T cells in atopic dermatitis and their possible role as peripheral biomarkers. Allergy.

[60] Batista D.I., Perez L., Orfali R.L., Zaniboni M.C., Samorano L.P., Pereira N.V., Sotto M.N., Ishizaki A.S., Oliveira L.M., Sato M.N. (2015). Profile of skin barrier proteins (filaggrin, claudins 1 and 4) and Th1/Th2/Th17 cytokines in adults with atopic dermatitis. J. Eur. Acad. Dermatol. Venereol..

[61] Seltmann J., Werfel T., Wittmann M. (2013). Evidence for a regulatory loop between IFN-γ and IL-33 in skin inflammation. Exp. Dermatol..

[62] Martel B.C., Dyring-Andersen B., Skov L., Thestrup-Pedersen K., Skov S., Skak K., Poulsen L.K. (2016). Different cytokine profiles of skin-derived T cell cultures from patients with atopic dermatitis and psoriasis. Inflamm. Res..

[63] Noda S., Suárez-Fariñas M., Ungar B., Kim S.J., de Guzman Strong C., Xu H., Peng X., Estrada Y.D., Nakajima S., Honda T. (2015). The Asian atopic dermatitis phenotype combines features of atopic dermatitis and psoriasis with increased TH17 polarization. J. Allergy Clin. Immunol..

[64] Tan Q., Yang H., Liu E.M., Wang H. (2017). Establishing a Role for Interleukin-17 in Atopic Dermatitis-Related Skin Inflammation. J. Cutan. Med. Surg..

[65] Wilson R.H., Whitehead G.S., Nakano H., Free M.E., Kolls J.K., Cook D.N. (2009). Allergic sensitization through the airway primes Th17-dependent neutrophilia and airway hyperresponsiveness. Am. J. Respir. Crit. Care Med..

[66] Cho J.S., Pietras E.M., Garcia N.C., Ramos R.I., Farzam D.M., Monroe H.R., Magorien J.E., Blauvelt A., Kolls J.K., Cheung A.L. (2010). IL-17 is essential for host defense against cutaneous Staphylococcus aureus infection in mice. J. Clin. Investig..

[67] Ma L., Xue H.B., Guan X.H., Shu C.M., Wang F., Zhang J.H., An R.Z. (2014). The Imbalance of Th17 cells and CD4(+) CD25(high) Foxp3(+) Treg cells in patients with atopic dermatitis. J. Eur. Acad. Dermatol. Venereol..

[68] Esaki H., Brunner P.M., Renert-Yuval Y., Czarnowicki T., Huynh T., Tran G., Lyon S., Rodriguez G., Immaneni S., Johnson D.B. (2016). Early-onset pediatric atopic dermatitis is TH2 but also TH17 polarized in skin. J. Allergy Clin. Immunol..

[69] Ness-Schwickerath K.J., Jin C., Morita C.T. (2010). Cytokine requirements for the differentiation and expansion of IL-17A- and IL-22-producing human Vgamma2Vdelta2 T cells. J. Immunol..

[70] Borghesi C., Nicoletti C. (1996). Autologous anti-idiotypic antibody response is regulated by the level of circulating complementary idiotype. Immunology.

[71] Vakil M., Sauter H., Paige C., Kearney J.F. (1986). In vivo suppression of perinatal multispecific B cells results in a distortion of the adult B cell repertoire. Eur. J. Immunol..

[72] Bogen B., Dembic Z., Weiss S. (1993). Clonal deletion of specific thymocytes by an immunoglobulin idiotype. EMBO J..

[73] Ghosh S.K., Chakrabarti D. (1993). Immunoregulation by processed immunoglobulin on B-cells. Indian J. Biochem. Biophys..

[74] Tan L., Fichtner A.S., Bruni E., Odak I., Sandrock I., Bubke A., Borchers A., Schultze-Florey C., Koenecke C., Förster R. (2021). A fetal wave of human type 3 effector γδ cells with restricted TCR diversity persists into adulthood. Sci. Immunol..

[75] Hunka J., Riley J.T., Debes G.F. (2020). Approaches to overcome flow cytometry limitations in the analysis of cells from veterinary relevant species. BMC Vet. Res..

[76] Bradford J.A., Buller G., Suter M., Ignatius M., Beechem J.M. (2004). Fluorescence-intensity multiplexing: Simultaneous seven-marker, two-color immunophenotyping using flow cytometry. Cytom. A.

[77] Kreslavsky T., von Boehmer H. (2010). gammadeltaTCR ligands and lineage commitment. Semin. Immunol..

[78] Jin Y., Xia M., Saylor C.M., Narayan K., Kang J., Wiest D.L., Wang Y., Xiong N. (2010). Cutting edge: Intrinsic programming of thymic γδT cells for specific peripheral tissue localization. J. Immunol..

[79] Muñoz-Ruiz M., Ribot J.C., Grosso A.R., Gonçalves-Sousa N., Pamplona A., Pennington D.J., Regueiro J.R., Fernández-Malavé E., Silva-Santos B. (2016). TCR signal strength controls thymic differentiation of discrete proinflammatory γδ T cell subsets. Nat. Immunol..

[80] Kabelitz D., Pechhold K., Bender A., Wesselborg S., Wesch D., Friese K., Janssen O. (1991). Activation and activation-driven death of human gamma/delta T cells. Immunol. Rev..

[81] Chen X., Zhang L., Tang S. (2019). MicroRNA-4497 functions as a tumor suppressor in laryngeal squamous cell carcinoma via negatively modulation the GBX2. Auris. Nasus. Larynx..

[82] Yang L., Hu Z., Jin Y., Huang N., Xu S. (2022). MiR-4497 mediates oxidative stress and inflammatory injury in keratinocytes induced by ultraviolet B radiation through regulating NF-κB expression. Ital. J. Dermatol. Venerol..

[83] Ji H., Li X.K. (2016). Oxidative Stress in Atopic Dermatitis. Oxid. Med. Cell Longev..

[84] Omata N., Tsukahara H., Ito S., Ohshima Y., Yasutomi M., Yamada A., Jiang M., Hiraoka M., Nambu M., Deguchi Y. (2001). Increased oxidative stress in childhood atopic dermatitis. Life Sci..

[85] Li Q.J., Chau J., Ebert P.J., Sylvester G., Min H., Liu G., Braich R., Manoharan M., Soutschek J., Skare P. (2007). miR-181a is an intrinsic modulator of T cell sensitivity and selection. Cell.

[86] Sonkoly E., Wei T., Janson P.C., Sääf A., Lundeberg L., Tengvall-Linder M., Norstedt G., Alenius H., Homey B., Scheynius A. (2007). MicroRNAs: Novel regulators involved in the pathogenesis of psoriasis?. PLoS ONE.

[87] Yew Y.W., Loh M., Thng S.T.G., Chambers J.C. (2020). Investigating causal relationships between Body Mass Index and risk of atopic dermatitis: A Mendelian randomization analysis. Sci. Rep..

[88] Guo Z., Yang Y., Liao Y., Shi Y., Zhang L.J. (2022). Emerging Roles of Adipose Tissue in the Pathogenesis of Psoriasis and Atopic Dermatitis in Obesity. JID Innov..

[89] He Y., Tsou P.S., Khanna D., Sawalha A.H. (2018). Methyl-CpG-binding protein 2 mediates antifibrotic effects in scleroderma fibroblasts. Ann. Rheum. Dis..

[90] Chang C.Y., Pasolli H.A., Giannopoulou E.G., Guasch G., Gronostajski R.M., Elemento O., Fuchs E. (2013). NFIB is a governor of epithelial-melanocyte stem cell behaviour in a shared niche. Nature.

