# Supplementary material for: IgG from Adult Atopic Dermatitis (AD) Patients Induces Nonatopic Neonatal Thymic Gamma–Delta T Cells (γδT) to Acquire IL-22/IL-17 Secretion Profile with Skin-Homing Properties and Epigenetic Implications Mediated by miRNA
Source: Int J Mol Sci. 2022 Jun 20;23(12):6872. doi: 10.3390/ijms23126872 (PMC9224404; doi:10.3390/ijms23126872)
Supplement: Supplementary file 1 [file ijms-23-06872-s001.zip › Supplementary Figures S1 to S4 Maj1.pdf]

Supplementary Figures S1 to S4

Title: IgG from adult atopic dermatitis (AD) patients induces non-atopic neonatal thymic gamma-delta T cells (γδT) to acquire IL-22/IL-17 secretion profile with skin-homing properties and epigenetic implications mediated by miRNA

Authors: Beatriz Oliveira Fagundes; Thamires Rodrigues de Sousa; Andrezza Nascimento; Lorena Abreu Fernandes; Fábio da Ressureição Sgnotto; Raquel Leão Orfali, MD, PhD[1]; Valéria Aoki, MD, PhD; Alberto José da Silva Duarte, MD, PhD, Sabri Saeed Sanabani, PhD, and Jefferson Russo Victor, PhD.

Figure S1

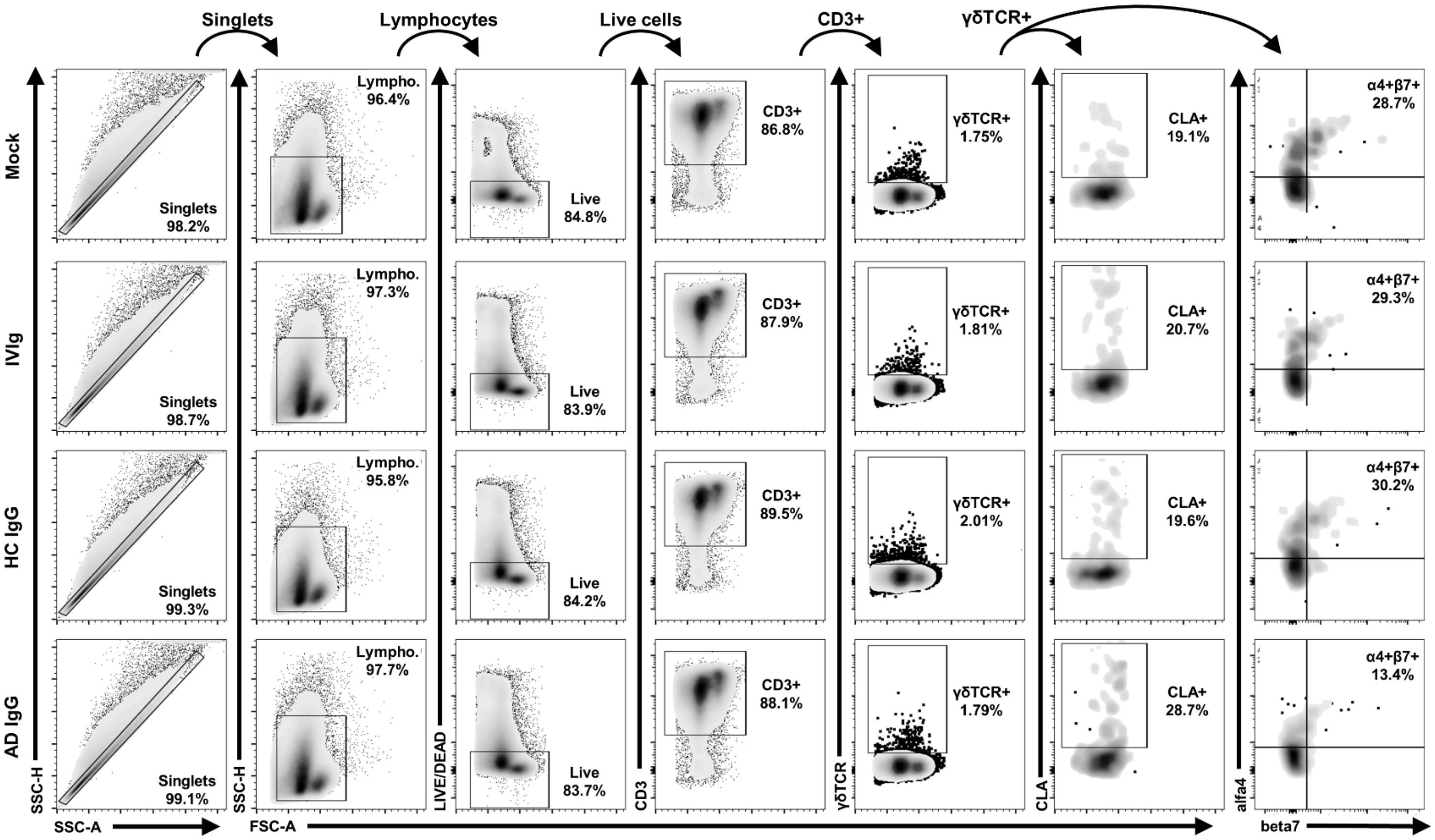

Figure S1: Illustrative dot plots of the gating strategy used to identify γδT cells and evaluate the expression of homing phenotype. Each sample was acquired by a consecutive approach using a single-cell gate (determined by SSC-A/SSC-H parameters), a lymphocyte gate (determined by relative SSC-A/FSC-A), a Live cells gate, a CD3+ cells gate, and a γδTCR gate to determine γδT (CD3+γδTCR+) cells. Samples were then acquired as CLA+ or α4+β7+. This figure illustrates the gating strategy used to determine γδT cells and its phenotype on mock, IVIg, HC IgG and AD IgG culture conditions (from upper to lower panels).

Figure S2

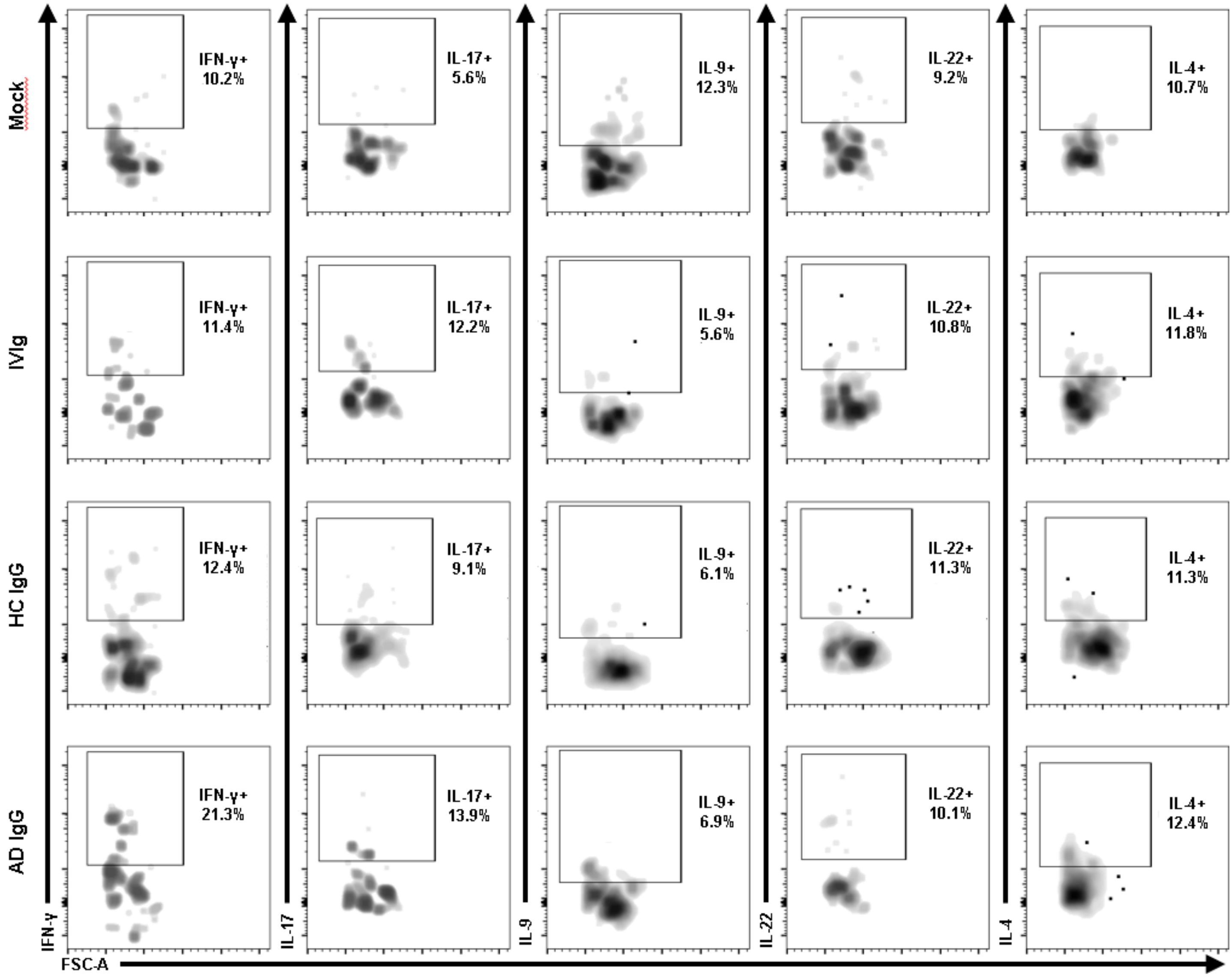

Figure S2: Illustrative dot plots of the gating strategy used to evaluate the production of cytokines on γδT cells after culture. Each sample was acquired by a consecutive approach using a single-cell gate (determined by SSC-A/SSC-H parameters), a lymphocyte gate (determined by relative SSC-A/FSC-A), a Live cells gate, CD3+ cells gate and γδTCR+ gating to determine γδT cells. Samples were then acquired as IFN-γ+, IL-17+, IL-19+, IL-22+ and IL-4+. This figure illustrates the gating strategy used to evaluate cytokines expression on mock, IVIg, HC IgG and AD IgG culture conditions (from left to right panels).

Figure S3

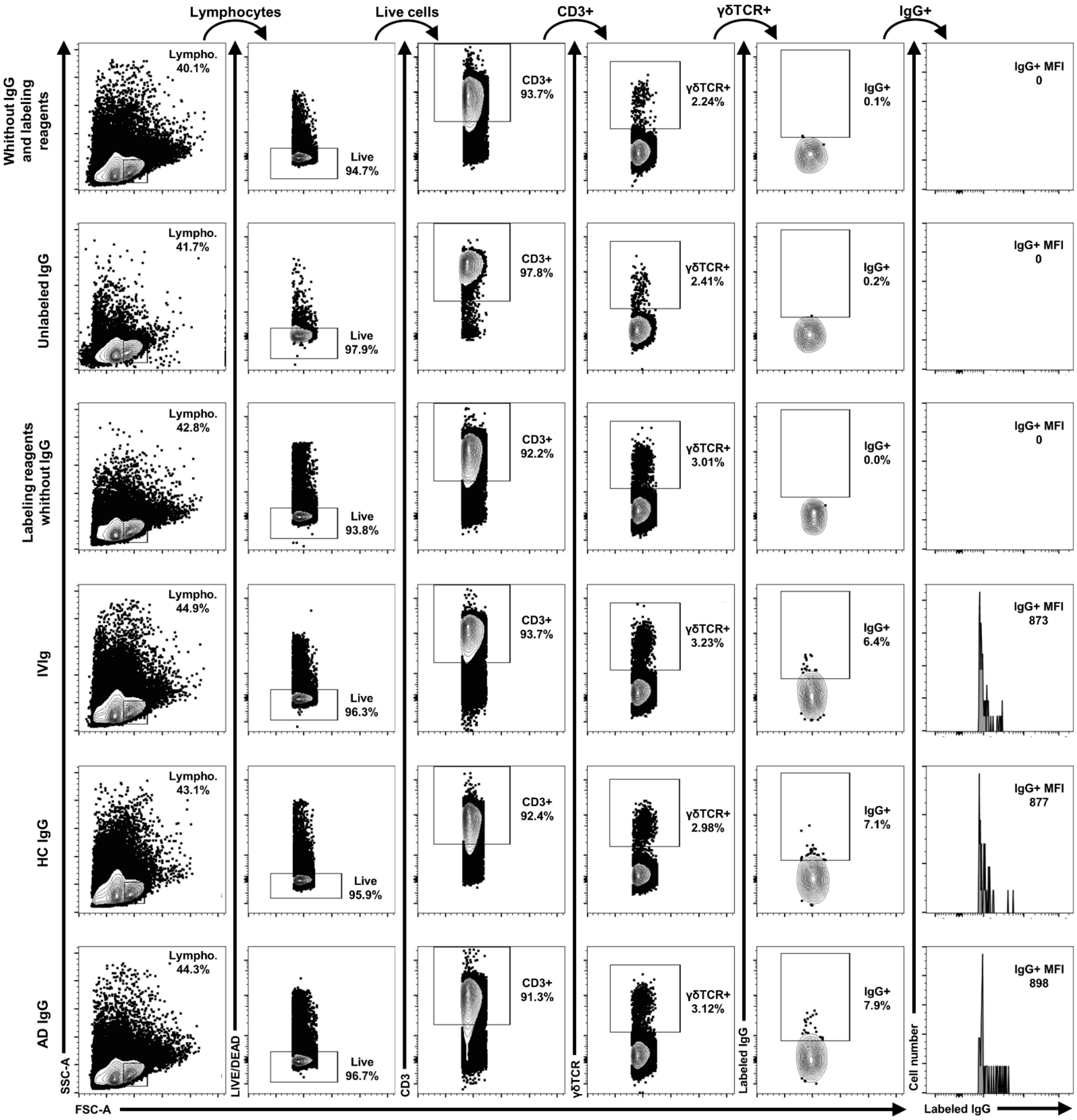

Figure S3: Illustrative dot plots of the gating strategy used to evaluate IgG-membrane interaction on γδT cells after culture. Each sample was acquired by a consecutive approach using a single-cell gate (determined by SSC-A/SSC-H parameters), a lymphocyte gate (determined by relative SSC-A/FSC-A), a Live cells gate, CD3+ cells gate and γδTCR+ gating to determine γδT cells. Samples were then acquired in the presence of Zenon human IgG labeling kit reagents but without IgG, in the presence of unlabeled AD IgG (IgG), in the presence of labeled IVIg, labeled IVIg HC IgG, or in the presence of labeled AD IgG. This figure illustrates the gating strategy used to detect IgG-membrane interactions in the cited conditions from upper to lower panels.

Figure S4

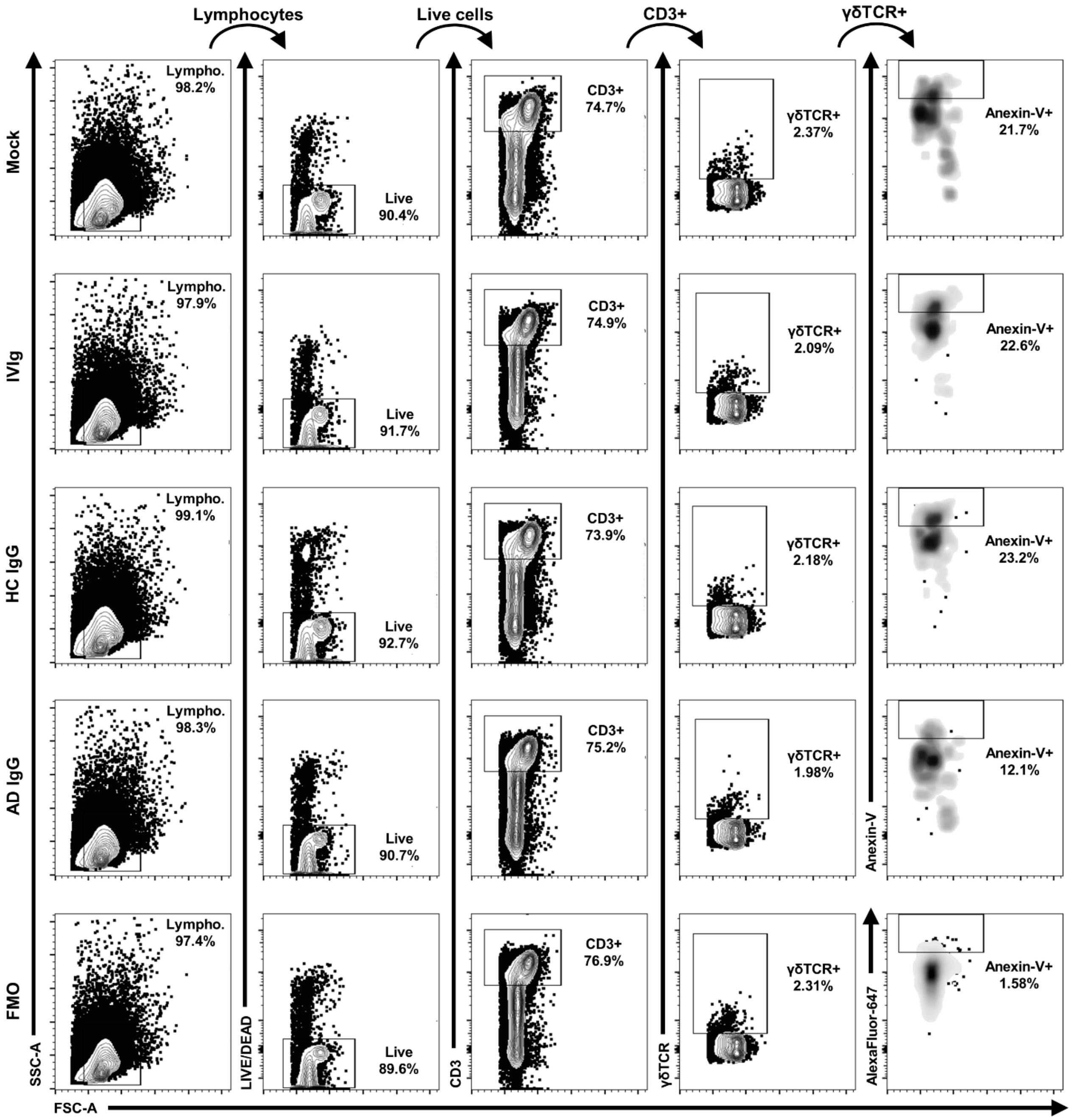

Figure S4: Illustrative dot plots of the gating strategy used to identify γδT cells and its staining with Anexin V. Each sample was acquired by a consecutive approach using a single-cell gate (determined by SSC-A/SSC-H parameters), a lymphocyte gate (determined by relative SSC-A/FSC-A), a Live cells gate, a CD3+ cells gate, and a γδTCR gate to determine γδT (CD3+γδTCR+) cells. Samples were then acquired as Anexin V+. This figure illustrates the gating strategy used to determine γδT cells and its Anexin V+ staining on mock, IVIg, HC IgG, AD IgG culture conditions. The lower panel demonstrate the Fluorescence minus one (FMO) gating strategy to precisely determine Anexin V gate.
